# Gene Expression Analysis Implicates a Death Receptor Pathway in Schizophrenia Pathology

**DOI:** 10.1371/journal.pone.0035511

**Published:** 2012-04-24

**Authors:** Vibeke Sørensen Catts, Cynthia Shannon Weickert

**Affiliations:** 1 Schizophrenia Research Laboratory, Schizophrenia Research Institute, Sydney, New South Wales, Australia; 2 Schizophrenia Research Laboratory, Neuroscience Research Australia, Sydney, New South Wales, Australia; 3 School of Biotechnology and Biomolecular Sciences, University of New South Wales, Sydney, New South Wales, Australia; 4 School of Psychiatry, University of New South Wales, Sydney, New South Wales, Australia; University of Illinois at Chicago, United States of America

## Abstract

An increase in apoptotic events may underlie neuropathology in schizophrenia. By data-mining approaches, we identified significant expression changes in death receptor signaling pathways in the dorsolateral prefrontal cortex (DLPFC) of patients with schizophrenia, particularly implicating the Tumor Necrosis Factor Superfamily member 6 (FAS) receptor and the Tumor Necrosis Factor [ligand] Superfamily member 13 (TNFSF13) in schizophrenia. We sought to confirm and replicate in an independent tissue collection the noted mRNA changes with quantitative real-time RT-PCR. To test for regional and diagnostic specificity, tissue from orbital frontal cortex (OFC) was examined and a bipolar disorder group included. In schizophrenia, we confirmed and replicated significantly increased expression of TNFSF13 mRNA in the DLPFC. Also, a significantly larger proportion of subjects in the schizophrenia group had elevated FAS receptor expression in the DLPFC relative to unaffected controls. These changes were not observed in the bipolar disorder group. In the OFC, there were no significant differences in TNFSF13 or FAS receptor mRNA expression. Decreases in BH3 interacting domain death agonist (BID) mRNA transcript levels were found in the schizophrenia and bipolar disorder groups affecting both the DLPFC and the OFC. We tested if TNFSF13 mRNA expression correlated with neuronal mRNAs in the DLPFC, and found significant negative correlations with interneuron markers, parvalbumin and somatostatin, and a positive correlation with PPP1R9B (spinophilin), but not DLG4 (PSD-95). The expression of TNFSF13 mRNA in DLPFC correlated negatively with tissue pH, but decreasing pH in cultured cells did not cause increased TNFSF13 mRNA nor did exogenous TNFSF13 decrease pH. We concluded that increased TNFSF13 expression may be one of several cell-death cytokine abnormalities that contribute to the observed brain pathology in schizophrenia, and while increased TNFSF13 may be associated with lower brain pH, the change is not necessarily causally related to brain pH.

## Introduction

Apoptosis is a cell death mechanism that can be triggered by normal developmental events or by extrinsic factors. Unbiased genome-wide analyses have implicated apoptotic pathways in the genetics of schizophrenia [1]. Other studies have linked polymorphisms and brain expression changes in apoptotic signaling genes to decreased calbindin-positive interneuron density and decreased number of perineuronal oligodendrocytes in schizophrenia [2]–[4]. Furthermore, an increased ratio of pro-apoptotic BAX to anti-apoptotic BCL-2 protein in the dorsolateral prefrontal cortex (DLPFC) from patients compared with controls suggests a bias favoring cell death processes in schizophrenia [5]. However, in this previous study there was no evidence for increased apoptosis in schizophrenia, as levels of the central apoptotic effector protease, caspase 3, were not changed [5]. The results of two other studies were also inconsistent with increased cell death. Reduced levels of the pro-apoptotic mitochondrial protein, Septin 4, and reduced density of apoptotic cells were found in DLPFC of people with schizophrenia [6]. Benes and colleagues [7] found fewer apoptotic cells in the anterior cingulate cortex in schizophrenia. Together these findings suggest apoptotic signaling may be altered in schizophrenia. However, it remains unclear whether cell death effectors are increased or decreased, and a direct link between apoptotic signaling and indices of cortical pathology has not been established.

Large changes in neuronal numbers, as would be predicted from ubiquitously altered apoptotic signaling, are not a core feature of schizophrenia [8], [9]. Rather neuronal density may be increased [10], [11] along with a reduction in somal size of neurons [12]–[17]. Therefore, if increased apoptotic signaling is etiologically involved in schizophrenia, presumably it may have more subtle effects on regression of subcellular components, such as dendritic spines, rather than cell death *per se*. Alternatively, increased cell death could affect a relatively small subset of neurons. Indeed, recent evidence suggests that certain types of cortical interneurons may be more susceptible to cell damage or death, such as fast-spiking parvalbumin [18] or layer II interneurons [19]. Currently, it is unclear if and how changes in cortical apoptotic signaling relate to the neuropathology found in two main cortical neuron populations, pyramidal cells and inhibitory interneurons, in schizophrenia.

In pyramidal neurons, schizophrenia pathology includes reduced dendritic fields [20]–[22] and loss of dendritic spines [23]–[25] affecting layer III across several cortical regions, but most commonly investigated in the DLPFC. Although pyramidal cell changes in schizophrenia may be secondary to a number of other processes [26], dendritic changes could be primary and result in neural system dysfunction. As reduced dendritic spine density has been directly linked to reduced excitatory inputs onto pyramidal neurons [27], it may account for a deficit in glutamatergic function in schizophrenia [28]. A possible explanation for reduced spine numbers in schizophrenia is increased localized apoptotic signaling in dendrites that results in retraction of spines [29], [30].

One of the most consistent findings in schizophrenia neuropathology is deficits in cortical inhibitory interneurons across multiple cortical regions [31], [32], though most commonly reported in DLPFC. Specifically, findings include reduced expression of the GABA-synthesizing enzyme glutamate decarboxylase 1, 67 kDa isoform (GAD_67_), which has recently been reviewed [33], reduced expression of the calcium binding protein parvalbumin [31], [34]–[41], and reduced expression of the neuromodulator peptide somatostatin [31], [37], [41], [42]. These interneuron-specific deficits could be primary or secondary due to reduced excitatory drive from pyramidal neurons [26]. One of the leading models of schizophrenia is based on the psychotomimetic effects of non-competitive antagonists of the N-methyl-D-aspartate (NMDA) receptor. Phencyclidine, ketamine and MK-801 block interneuron function in proportion to their NMDA blocking potency [43]. While the data to support altered rates of cell death of interneuron subtypes in cortex from patients with schizophrenia are lacking [44], a study of gene expression in hippocampal interneurons has implicated the apoptosis-related cell cycle regulator histone deacetylase 1 in this disease [45], [46]. Since many different ligands, receptors and effectors are involved in regulating cell death [47] we took a broad approach to surveying multiple cell death transcripts that might be involved in schizophrenia in order to identify a specific cell death pathway for more in-depth analysis.

In the current study, we explored the microarray database from the Stanley Medical Research Institute (SMRI) Array postmortem brain tissue collection [48] as a first step to identify apoptotic pathways with altered gene expression in the DLPFC from patients with schizophrenia. Significant expression changes in mRNA encoding molecules within three overlapping death receptor signaling categories were noted. In common, the three categories implicated the Tumor Necrosis Factor Superfamily member 6 (FAS) receptor or its putative ligand, Tumor Necrosis Factor (ligand) Superfamily, member 13 (TNFSF13) [49] and the down-stream cytoplasmic BH3 interacting domain death agonist (BID). In the second stage of this study, we sought to confirm by quantitative real time PCR (qRT-PCR) the altered mRNA levels in cell-death genes in the DLPFC SMRI Array Collection. We then carried out a replication study in an independent DLPFC tissue collection from the Australian Tissue Resource Collection (TRC) using qRT-PCR. The DLPFC SMRI Array Collection included a bipolar disorder group enabling investigation of diagnostic specificity of gene expression changes. Additionally, we measured TNFSF13, the FAS receptor and BID mRNA levels in the orbital frontal cortex (OFC) to assess regional specificity. We next examined the relationships between mRNA levels of TNFSF13 and markers of known neuropathology, i.e. expression levels of interneuron (parvalbumin, somatostatin) and dendritic spine (PPP1R9B and DLG4) mRNAs in the TRC collection. Analysis of demographic variables and gene expression revealed a large and significant negative correlation between tissue pH and TNFSF13 expression. Since steady state levels of most transcripts are typically decreased with lower tissue pH [50], [51], the negative correlation with TNFSF13 and pH was unexpected and suggested that an active process of gene induction might be occurring in the brain during low pH conditions. We therefore supplemented our postmortem findings by performing two *in vitro* cell culture studies to investigate experimentally the relationship between intracellular pH and TNFSF13.

## Results

### Pathway analysis of the SMRI Array database

**Figure 1:**
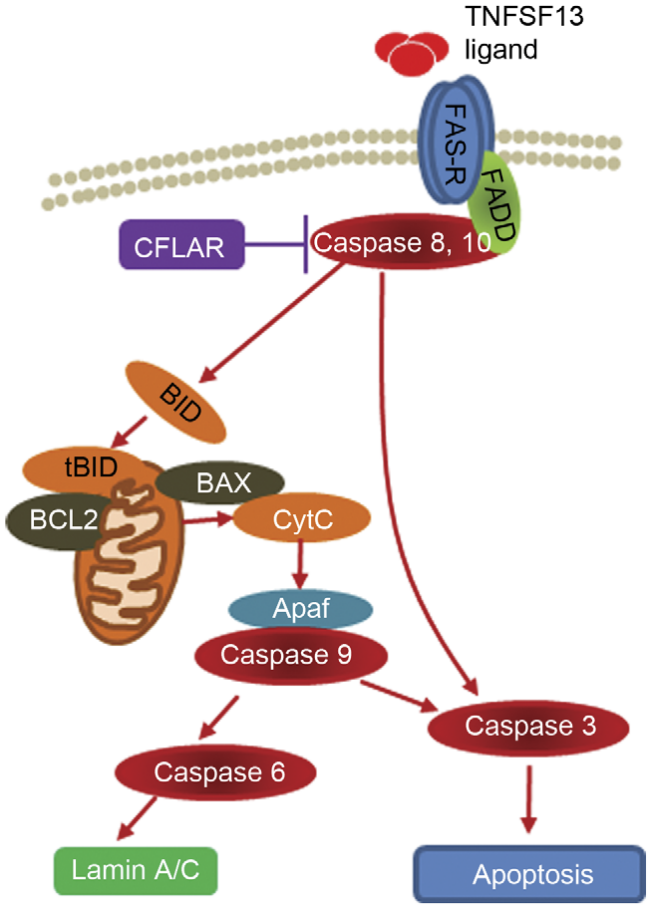
FAS receptor pathway in peripheral cells [**76**]. The membrane FAS-R (blue) forms a trimer upon ligand binding (TNFSF13, red). The adaptor molecule FADD (green) then binds to the death domain of FAS-R through its own death domain. The amino terminus of FADD facilitates binding to caspase-8 (maroon oval). Caspase-8 self-activates through proteolytic cleavage into active caspase subunits, which can cleave other effector caspases (3, 6, 9), eventually leading to DNA degradation, membrane blebbing, and other hallmarks of apoptosis. In most cell types (Type II), caspase-8 catalyzes the cleavage of the pro-apoptotic BH3-only protein BID (orange) into its truncated form, tBID. tBID engages anti-apoptotic members of the family (e.g. BCL-2), allowing pro-apoptotic members (e.g. BAX) to translocate to the outer mitochondrial membrane, thus permeabilizing the membrane and facilitating release of pro-apoptotic proteins such as cytochrome c (tan). Cytochrome c release reinforces the cellular apoptotic drive through the intrinsic apoptotic pathway. Death receptors 4/5 (TNFSF10) appear to have identical intracellular pathway signaling to FAS receptor [76].

Seventeen apoptotic pathways of potential interest were identified (see Table S1). Nine of these pathways had altered expression of at least 1 gene. When a heat map ranking pathways by GO term enrichment was consulted, we noted significant differences in death receptor signaling pathways, FAS receptor, death receptor 3 and death receptor 4/5. Within these death receptor signaling pathways, 6 molecules (Table S1) had altered expression in the DLPFC of patients with schizophrenia. Five of these, TNFSF13 ligand (1.10 fold increase, p = 0.0114), FAS receptor (1.05 fold increase, p = 0.0014), CFLAR (1.05 fold increase, p = 0.0343), BID (−1.05 fold decrease, p = 0.0288) and Lamin A/C (1.07 fold increase, p = 0.0001), were selected for further analysis as these death molecules interact with each other (outlined in Figure 1).

### qRT-PCR analysis of TNFSF13-FAS receptor pathway genes in the DLPFC

We used qRT-PCR with TaqMan probes designed to capture all known expressed variants of the selected genes (pan, Table 1) to confirm and replicate the changes observed by others using microarray analysis in the SMRI Array collection of DLPFC tissue. Initial qRT-PCR pilot experiments indicated that Lamin A/C was expressed at very low levels in the DLPFC. We therefore present results for four apoptotic pathway transcripts, describing the results obtained for the DLPFC using the SMRI collection, the TRC collection and the combined collections for each transcript in turn. Combining the collections was considered useful as the distribution of the data was better appreciated, more accurate effect sizes could be calculated, and because it was justified on the grounds of comparability of means and variance of expression data.

**Table 1:** Genes analyzed with ABI Taqman Gene Expression assay part numbers.

Gene symbol	Gene name	Taqman assay
TNFSF13	Tumor necrosis factor (ligand) superfamily, member 13	Hs00182565_m1
FAS	Fas (TNF receptor superfamily, member 6)	Hs00236330_m1
CFLAR	CASP8 and FADD-like apoptosis regulator	Hs01116280_m1
CFLAR	CASP8 and FADD-like apoptosis regulator	Hs00153439_m1
BID	BH3 interacting domain death agonist	Hs00609632_m1
Lamin A/C	Lamin A/C	Hs00153462_m1
DLG4	discs, large homolog 4	Hs00176354_m1
PPP1R9B	protein phosphatase 1, regulatory (inhibitor) subunit 9B	Hs00261636_m1
SST^1^	Somatostatin	Hs00356144_m1
PV^1^	Parvalbumin	Hs00161045_m1
ACTB	β-Actin	Hs99999903_m1
TBP	TATA box binding protein	Hs00427620_m1
UBC	Ubiquitin C	Hs00824723_m1

1 Data previously published by Fung *et al.* [41].

**Figure 2:**
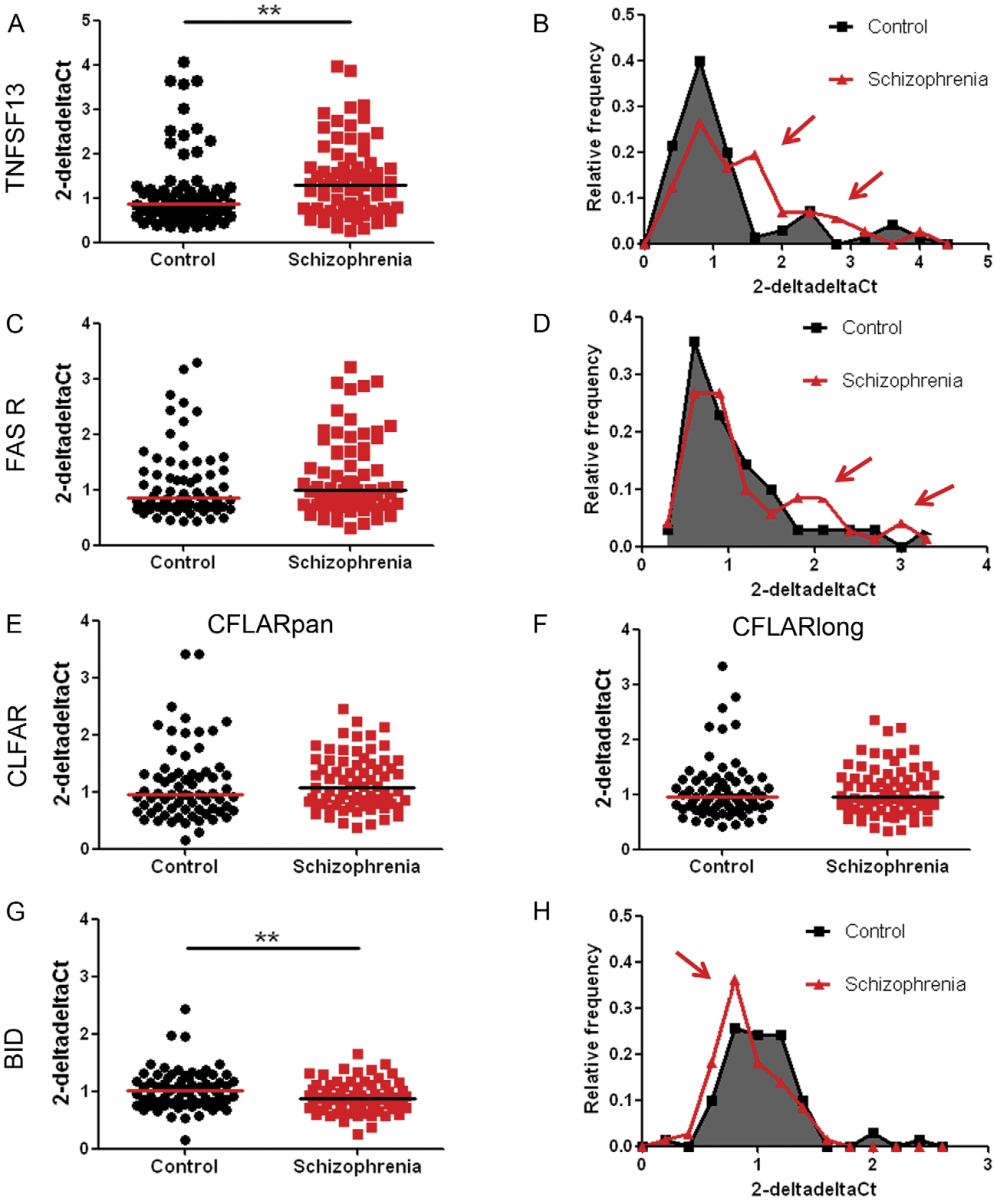
Gene expression levels of TNFSF13-FAS receptor pathway genes of interest relative to three housekeeping genes in dorsolateral prefrontal cortex from patients with schizophrenia. cDNA samples obtained from the combined SMRI and NSW TRC collections from individuals with schizophrenia and unaffected controls were subjected to qRT-PCR. Expressions of TNFSF13 (A), FAS receptor (C), CFLAR_pan_ (E), CFLAR_long_ (F) and BID (G) relative to three housekeeping genes (β-actin, TATA box binding protein and ubiquitin C) were calculated using the deltadelta Ct method. Horizontal lines indicate the population median, except for panel (G) where they indicate the mean as those data were normally distributed. Frequency distributions for TNFSF13, FAS receptor and BID are displayed in panels (B), (D), and (H). ** p<0.01.

**Figure 3:**
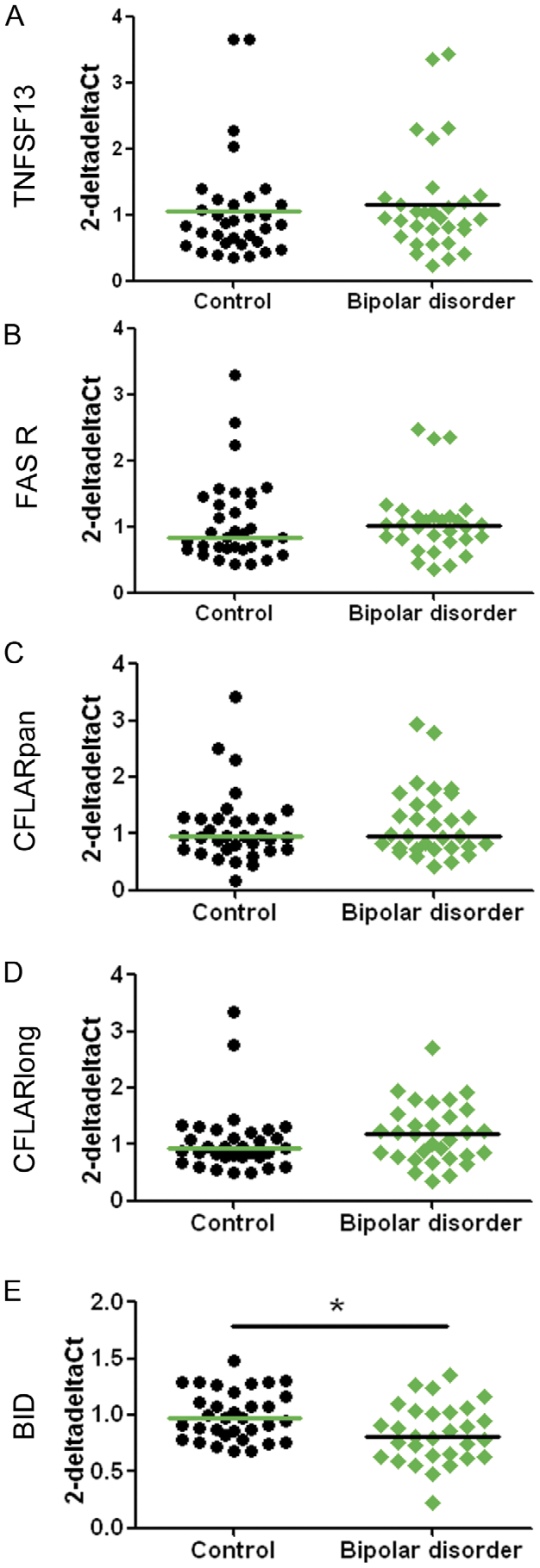
Gene expression levels of TNFSF13-FAS receptor pathway genes of interest relative to three housekeeping genes in dorsolateral prefrontal cortex from patients with bipolar disorder. cDNA samples obtained from the SMRI collection from individuals with bipolar disorder and unaffected controls were subjected to qRT-PCR. Expressions of TNFSF13 (A), FAS receptor (B), BID (C), CFLAR_pan_ (D), and CFLAR_long_ (E) relative to three housekeeping genes (β-actin, TATA box binding protein and ubiquitin C) were calculated using the deltadelta Ct method. Horizontal lines indicate the population median, except for panel (E) where they indicate the mean as those data were normally distributed. * p<0.05.

We found that the expression of TNFSF13 mRNA in the DLPFC was significantly increased in patients with schizophrenia in both the SMRI (Figure S1, panel A, U = 442.00, p = 0.048 one-tailed) and the NSW TRC (Figure S1, panel B, U = 463.00, p = 0.0125 one-tailed) collections. In the combined collections TNFSF13 mRNA was increased by 27% in patients with schizophrenia relative to unaffected controls (Figure 2A: U = 1824.00, p = 0.003 one-tailed, effect size r = 0.23). While individuals in both the control and schizophrenia group could express elevated TNFSF13 mRNA, in the combined collection we noted that more patients with schizophrenia fell into the high expression range as illustrated by frequency distribution plots (see red arrows in Figure 2B). This was significant when high TNFSF13 expression was defined as 2-deltadelta Ct values greater than the median plus 1.25 times the interquartile range for the unaffected control group (>1.5787: χ2(1) = 4.931, p = 0.026, two-tailed). On average the mean of high TNFSF13 expressors in the control group (3.053±0.5615) were 5 standard deviations away from the mean of those TNFSF13 mRNA values that formed a normal distribution (0.8919±0.4308: D(62) = 0.107, p = 0.073). Patients with schizophrenia were 2.47 times more likely to have high TNFSF13 expression than unaffected controls. There was no evidence of a changed expression of TNFSF13 mRNA in the bipolar group relative to unaffected controls in the DLPFC (Figure 3A: U = 457.00, p = 0.464 two-tailed, effect size r = 0.09).

The group-wise expression of FAS receptor was not statistically significantly increased in patients with schizophrenia in the SMRI collection (Figure S1, panel C, U = 501.00, p = 0.174 one-tailed) or the NSW TRC collection (Figure S1, panel D, U = 554.00, p = 0.145 one-tailed), or within the combined DLPFC collections (Figure 2C, U = 2105.00, p = 0.08 one-tailed, effect size r = 0.12). However, we once again noted that more patients with schizophrenia fell in the high expression range (red arrows in Figure 2D). This was significant when high FAS receptor expression was defined as 2-deltadelta Ct values greater than the median plus 1.25 times the interquartile range for the unaffected control group (>1.661: χ2(1) = 4.875, p = 0.027, two-tailed, effect size r = 0.19). On average the mean of high FAS receptor mRNA expressors in the control group (1.811±0.585) were 5 standard deviations away from the mean of those FAS receptor mRNA values that formed a normal distribution (0.758±0.193: D(48) = 0.126, p = 0.056). Patients with schizophrenia were 2.61 times more likely to have high FAS receptor expression than unaffected controls. FAS receptor expression in the bipolar disorder group was not significantly different to that of unaffected controls (Figure 3B: U = 459.00, p = 0.62 two-tailed, effect size r = 0.06).

CFLAR_pan_ expression was not significantly changed within the schizophrenia group in the SMRI or NSW TRC collections (Figure S1, panels E–F), nor in the combined collections (U = 2174.00, p = 0.101 one-tailed, Figure 2E, effect size r = 0.11). CFLAR is expressed as several different protein forming splice variants [52], the most important ones being CFLAR_short_ and CFLAR_long_, which have opposing effects on caspase-8 activity. While it is not possible to specifically target CFLAR_short_ transcripts using qRT-PCR, we determined expression of CFLAR_long_ and found it not to be differentially expressed in the schizophrenia group in the SMRI or NSW TRC collections (Figure S1, panels G–H), nor in the combined collections (U = 2407.00, p = 0.323 one-tailed, Figure 2F, effect size r = 0.04). Similarly, there were no group differences between patients with bipolar disorder and unaffected controls (Figure 3C, D) in CFLAR_pan_ (U = 413.00, p = 0.186 two-tailed, effect size r = 0.09) or CFLAR_long_ expression (U = 457.00, p = 0.0.464 two-tailed, effect size r = 0.16).

The expression of the pro-apoptotic gene, BID was significantly decreased in DLPFC from the SMRI collection (t(66) = 2.381, p = 0.01 one-tailed, Figure S1, panel I), but not in the NSW TRC (t(71) = 1.607, p = 0.057 one-tailed, Figure S1, panel J). In the combined collection, the decreased expression of BID in tissue from patients with schizophrenia was statistically significant (Figure 2G, H: t(139) = 2.656, p = 0.005 one-tailed, effect size r = 0.22). Patients with bipolar disorder also had reduced expression of BID (Figure 3E: t(61) = 2.74, p = 0.005 one-tailed, effect size r = 0.33).

### qRT-PCR analysis of TNFSF13-FAS receptor pathway genes in the OFC

**Figure 4:**
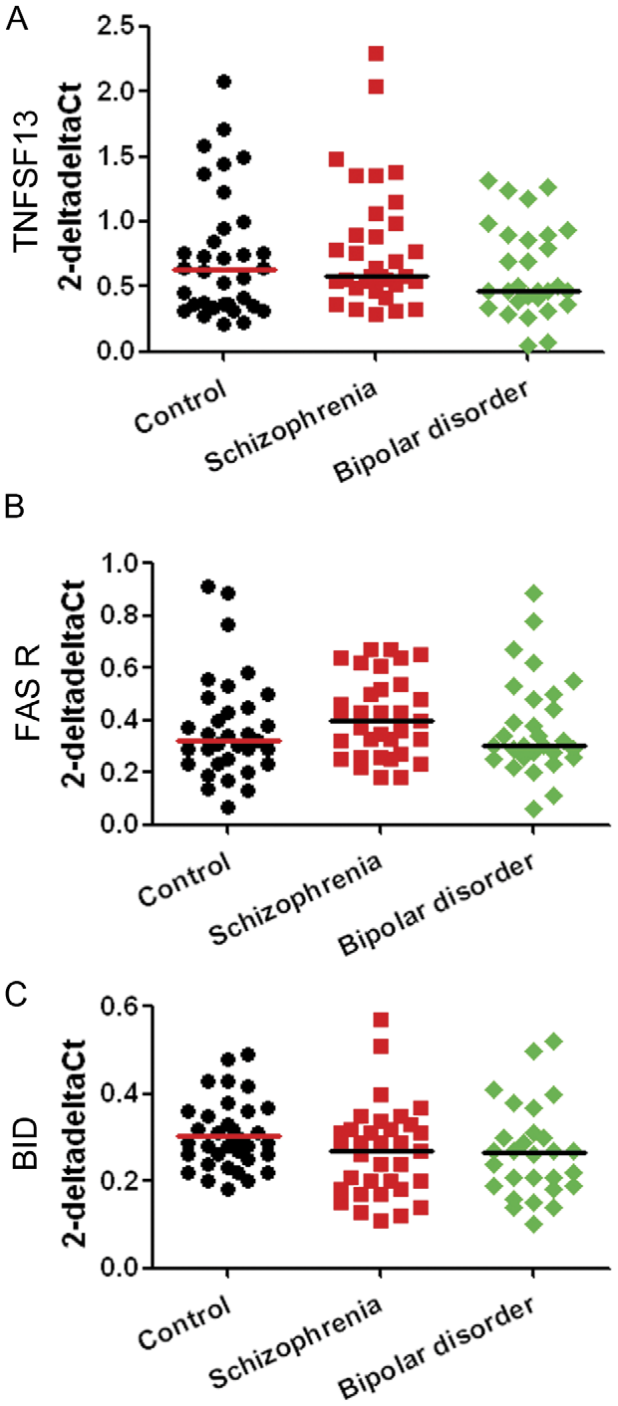
Gene expression levels of TNFSF13, FAS receptor and BID relative to three housekeeping genes in OFC from patients with schizophrenia, patients with bipolar disorder and unaffected controls. cDNA samples from individuals with schizophrenia, bipolar disorder and unaffected controls were subjected to qRT-PCR. Expressions of TNFSF13 (A), FAS receptor (B) and BID (C) relative to three housekeeping genes (β-actin, TATA box binding protein and ubiquitin C) were calculated using the deltadelta Ct method. Horizontal lines indicate the population median, except for panel (C) where they indicate the mean as those data were normally distributed.

We observed no significant effect of diagnosis on mRNA levels of TNFSF13 (χ^2^(2) = 2.38, p = 0.304), FAS receptor (χ^2^(2) = 2.15, p = 0.342), or BID (F(2) = 1.675, p = 0.193) in the OFC of the SMRI collection (Figure 4A, B, C). The effect size between control and schizophrenia cases for TNFSF13 in the OFC (r = 0.10) suggests that this negative finding is not simply attributable to the smaller sample size within the SMRI collection relative to that of the combined collections. The effect size for BID between controls and schizophrenia cases (r = 0.32) and bipolar disorder cases (r = 0.34) indicated that diagnosis accounted for over 10% of the variance in gene expression within either diagnostic group.

### TNFSF13 expression in the DLPFC and its relationship to pyramidal cell and interneuron markers

We measured expression of two dendritic spine mRNAs in the TRC collection, but failed to observe any altered transcript levels in patients with schizophrenia relative to controls for PPP1R9B (U = 609.00, p = 0.529) or DLG4 (t(71) = −1.139, p = 0.258). The expression levels of parvalbumin and somatostatin have previously been reported to be reduced in patients with schizophrenia in the TRC collection [41]. To explore the relationship between TNFSF13 expression and markers of pyramidal cell spines (PPP1R9B and DLG4) and interneuron subtypes (parvalbumin and somatostatin), we calculated the observed variances between these measures (Table 2). This revealed significant negative correlations between TNFSF13 mRNA and parvalbumin and somatostatin mRNAs. TNSFSF13 was positively correlated with PPP1R9B, but there was only a weak relationship with DLG4 mRNA, where TNFSF13 accounted for less than 10% of the variance. As pH correlated negatively with the expression of TNFSF13 mRNA, we next carried out regression analyses including pH to determine its contribution to the observed association between TNFSF13 and spine and interneuron markers. We found that in the control group pH accounted for 38% of the variance of somatostatin, and 11% of DLG4. pH accounted for significant amounts of variance in parvalbumin (12%), somatostatin (36%), DLG4 (13%) and PPP1R9B (11%) in the schizophrenia group. Over and above the effect of pH, TNFSF13 expression accounted for significant variance in PPP1R9B (24–36%) in both groups, however TNFSF13 mRNA did not account for any additional variance in the two interneuron mRNA measures.

**Table 2:** Variance (R^2^) in parvalbumin, somatostatin, PPP1R9B and DLG4 mRNA transcript levels accounted for by TNFSF13 expression and tissue pH in the NSW TRC collection.

		Unaffected control group			Schizophrenia group		
		TNFSF13	pH	Δ TNFSF13	TNFSF13	pH	Δ TNFSF13
Interneurons							
Parvalbumin	R^2^	.170*	.051	.058	.072	.123*	.003
	β	−.413*	.225		−.269	.350*	
Somatostatin	R^2^	.293**	.383***	.006	.247**	.364***	.020
	β	−.541***	.619***		−.497**	.604***	
Spines							
PPP1R9B	R^2^	.169*	.085	.244**	.451***	.112*	.358***
	β	.411*	−.262		.672***	−.335*	
DLG4	R^2^	.063	.111*	.003	.002	.127*	.126*
	β	−.251	.333*		.049	.357*	

Δ TNFSF13: Change in R^2^ accounted for by TNFSF13 when pH controlled for.

β Standardized coefficient.

* p<0.05;

** p<0.01;

*** p<0.001.

Our analysis of the relationship of TNFSF13 pathway gene expressions in the DLPFC with demographic and clinical variables (Table 3) revealed significant negative correlations with tissue pH. Tissue pH also appeared to play a significant role in the relationship between TNFSF13 and markers of interneuron health. This led us to focus our next set of studies on the role of tissue pH in TNFSF13 expression.

**Table 3:** Relationship of gene expression in the DLPFC with demographic and clinical variables.

	TNFSF13	FAS receptor	CFLAR_pan_	CFLAR_long_	BID
Age	n.s.	n.s.	r = 0.331, p = 0.008	n.s.	n.s.
Sex	n.s.	n.s.	n.s.	n.s.	n.s.
Brain hemisphere	n.s.	n.s.	n.s.	U = 0.409, p = 0.007^a^	n.s.
Postmortem interval	r = −0.173, p = 0.023	r = −0.247, p = 0.001	n.s.	n.s.	n.s.
pH	r = −0.484, p<0.001	r = −0.404, p<0.001	r = −0.410, p<0.001	r = −0.385, p<0.001	n.s.
Smoking	n.s.	n.s.	n.s.	n.s.	t(55) = 2.17, p = 0.035^b^
Age at onset	n.s.	n.s.	n.s.	n.s.	n.s.
Duration of illness	n.s.	n.s.	r = 0.280, p = 0.007	n.s.	n.s.
Lifetime antipsychotic use	r = 0.289, p = 0.014	n.s.	r = 0.238, p = 0.044	n.s.	n.s.
Antidepressant	n.s.	n.s.	n.s.	n.s.	n.s.
Death by suicide	U = 268, p = 0.0027	n.s.	n.s.	n.s.	n.s.

All correlations listed are Spearman's.

n.s. Not statistically significant.

a Increased in right hemisphere.

b Decreased in patients with schizophrenia who smoke.

### Cell culture studies of the relationship between TNFSF13 and FAS receptor expression and pH

We tested experimentally whether lowered intracellular pH would increase TNFSF13 mRNA levels in cultured glioblastoma cells, U-87 MG. Because statistical correlations in postmortem tissue do not indicate directional cause, we also determined if higher levels of TNFSF13 could lead to lower pH in U-87 MG cell cultures.

**Figure 5:**
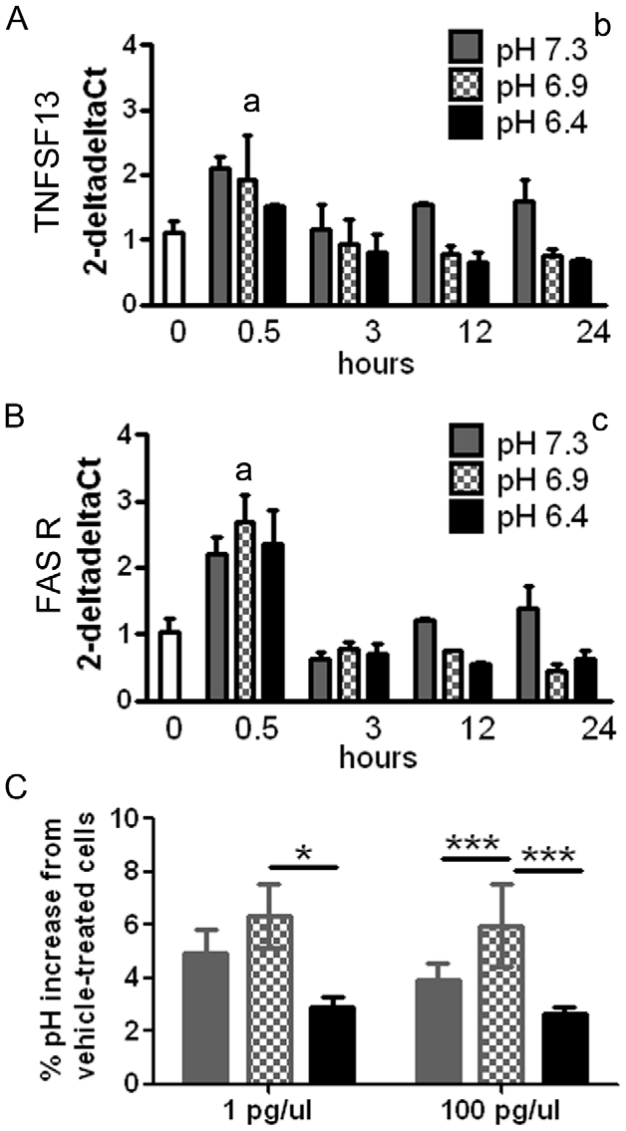
The effect of TNFSF13 exposure on intracellular pH, and of decreased intracellular pH on TNFSF13 and FAS receptor gene expression. Intracellular pH of U-87 MG cells was artificially decreased using nigericin and phosphate buffer to pH 6.4, 6.9 and 7.3 for 0.5, 3, 12 or 24 hours and transcript levels of the TNFSF13 (A) or FAS receptor (B) genes measured by qRT-PCR. Expression levels were calculated relative to three housekeeping genes (β-actin, TATA box binding protein and ubiquitin C) using the deltadelta Ct method. ^a^Transcript levels at the 0.5 hours time point were significantly greater than at all other time points, ^b^Decreasing pH significantly suppressed transcript levels of TNFSF13, ^c^There was no significant effect of pH on FAS receptor transcripts. U-87 MG glioblastoma cell cultures were treated with TNFSF13 (1 pg or 100 pg TNFSF13/microliter of culture media) for up to 48 hours and the intracellular pH determined (C). *p<0.05, ***p<0.001.

In the first study, we lowered intracellular pH by exposing cells to nigericin and potassium phosphate buffers and then determined expression of TNFSF13 and FAS receptor mRNAs 0.5, 3, 12 and 24 hours later. In contrast to our hypothesis, we found that cells with reduced pH (pH 6.4 and 6.9) had reduced TNFSF13 mRNA expression relative to cells with physiological pH (F(2,23) = 4.464, p = 0.023 two-way ANOVA, post-hoc tests p<0.05 for both pH 6.4 and 6.9, Figure 5A). While a similar expression pattern was observed for the FAS receptor (Figure 5B), the two-way ANOVA did not support a significant effect of pH on this transcript (F(3,23) = 1.616, p = 0.220). There was a significant effect of time on expression of both transcripts (TNFSF13: F(3,23) = 4.937, p = 0.009; FAS receptor: F(3,23) = 41.263, p<0.001) attributable to the expressions at the 0.5 hour time point being greater than the 3, 12, and 24 hour time points (post-hoc tests p<0.05). Although exposure to nigericin caused an early increase in TNFSF13 and FAS receptor expression (0.5 hour time point) this increase occurred irrespective of the culture media pH.

In the second study, we examined whether increased levels of TNFSF13 ligand could lower intra-cellular pH. This was done by adding TNFSF13 to glioblastoma cell cultures (U-87 MG: Figure 5C). Again, contrary to our hypothesis, we found that intracellular pH was significantly increased relative to vehicle-treated cells 12, 24 and 48 hours following exposure to TNFSF13 (t(5) = 5.180–10.38, p<0.01 for all time points, one-sample t-test uncorrected for multiple comparisons). The highest pH was measured 24 hours following TNFSF13 exposure with evidence of a return towards baseline at 48 hours.

## Discussion

The current study is the first to specifically report, confirm, and replicate in an independent postmortem tissue collection, an increase in mRNA transcript levels of the tumor necrosis factor receptor ligand, TNFSF13, in the DLPFC of patients with schizophrenia. The replication of this finding in an independent tissue collection (NSW TRC) and the magnitude of the TNFSF13 expression change suggest the observed increase is unlikely to be due to Type I error. The study is also the first study to provide direct evidence of a relationship between altered apoptotic pathway signaling and putative neuronal markers of neuropathologies of schizophrenia. The increase in TNFSF13 mRNA was not evident in the OFC of patients with schizophrenia suggesting that increased TNFSF13 expression in the DLPFC may not be a non-specific consequence of severe mental illness [3]. Whilst other studies have observed increased TNFSF13 expression in reactive astrocytes in multiple sclerosis [53] and in cells surrounding tumor tissue [54], the absence of TNFSF13 expression changes in patients with bipolar disorder suggests at least a degree of diagnostic specificity for the TNFSF13 mRNA change between the two psychiatric groups. The robust abnormality in TNFSF13 mRNA transcript levels in the DLPFC warrants confirmation at the protein level as well as further study of factors contributing to the increased TNFSF13 expression in patients with schizophrenia.

TNFSF13 has been shown to bind to four tumor necrosis factor receptor family members [49], [55]. However, the expressions of three of these receptors are very limited or entirely absent in the CNS [55], [56] and were therefore not pursued in the current study. The fourth receptor, FAS, was originally identified as a lymphocyte receptor but is also widely expressed in the CNS [57]. Consistent with TNFSF13 activating the FAS receptor pathway, we found that TNFSF13 transcript levels correlated strongly with FAS receptor mRNA expression and that patients with schizophrenia were more likely to have high FAS receptor expression (39%) in the DLPFC as compared to controls (15% with increased FAS receptor).

Ligand binding to FAS receptor typically results in the formation of a death-inducing signaling complex, of which CFLAR is an important modulating component [58]. Our qRT-PCR investigation did not confirm or replicate the increased CFLAR expression observed in schizophrenia tissue by microarray. Inability to confirm array results may be attributable to low transcript levels of CFLAR or failure of the qRT-PCR probe to capture the same transcript(s) as the microarrays [59]. Despite CFLAR transcripts levels being relatively low in the CNS, we found CFLAR probes amplified robustly at the same cDNA concentration as TNFSF13 and FAS receptor probes. This suggests lack of qRT-PCR confirmation of array results for CFLAR in our study is most likely attributable to differences in the transcripts captured by the different assays. Pinpointing differences in transcripts captured by array compared to our qRT-PCR will take further transcript characterization studies.

Increased TNFSF13 expression suggests increased apoptotic signaling in our schizophrenia group. However, as predicted by the results in the SMRI array database we found decreased transcripts levels of the pro-apoptotic BID in patients with schizophrenia. Decreased gene expression could be a compensatory change to counteract detrimental effects of increased apoptotic signaling, however, the absence of a negative correlation between TNFSF13 mRNA expression and BID mRNA does not support a direct relationship between the two transcripts. Decreased BID transcript levels in the DLPFC were also observed in patient with bipolar disorder and thus not specific to just one psychotic disorder. Because expression of other BH3-genes, such as BAX and BCL-2, has been observed to be regulated by several different antidepressants and mood stabilizers [60] commonly prescribed to both patients with schizophrenia and bipolar disorder, we explored but did not find support for antidepressant medications playing a role in the decreased expression of BID in our patient groups. There were more patients who were smokers in the schizophrenia groups than in the unaffected control group. We found that smoking might interact with disease-specific factors in the schizophrenia group and contribute to decreased BID expression, a phenomena described previously for other transcripts [61].

In order to put changes in TNFSF13 mRNA levels in the context of known schizophrenia neuropathology, we explored the relationship between levels of TNFSF13 mRNA and transcript levels of interneuron markers, somatostatin and parvalbumin, as well as spine markers, DLG4 and PPP1R9B. Our regression analysis results are consistent with TNFSF13 contributing to the established interneuron deficits in schizophrenia. As parvalbumin mRNA levels in schizophrenia tissue have been observed to correlate positively with pH in several studies [36], [41], [62], [63] and TNFSF13 expression correlated negatively with tissue pH in the current study, this means that as tissue pH is lowered, parvalbumin mRNA decreases but TNFSF13 mRNA increases. Our observation that variance in tissue pH could account for the relationship between interneuron markers and TNFSF13 mRNA is significant because it suggests that an active process (upregulation of TNFSF13) related to tissue pH may be a mediating factor of the interneuron deficit. While we did not observe any effect of diagnostic group on the expression levels of either spine marker, DLG4 and PPP1R9B, this was not entirely unexpected as previous reports have also failed to find change in these mRNAs in schizophrenia [64], [65]. This suggests that DLG4 and PPP1R9B mRNA levels may not closely reflect the morphological changes observed in dendritic spines in patients with schizophrenia. Given that changes in spine marker mRNA may be more prevalent in some cases as compared to others, we asked if levels of spine markers relate to the observed changes in TNFSF13 expression by regression analysis. We hypothesized that increased TNFSF13 would be related to decreased spine markers. Surprisingly, our result suggests that increased TNFSF13 expression may contribute to increased PPP1R9B expression and that this effect is independent of pH. However, our cell culture results indicate that ligands such as TNFSF13 can transiently increase pH not decrease it as found in human brain tissue. Perhaps in vivo manipulation of TNFSF13 in brain may better capture the interplay between different cell types. Our experimental cell culture studies did suggest that changes in TNFSF13 and pH may not be directly linked, but that since they occur in the context of brain tissue may both represent reactions to another upstream event.

The negative correlation between TNFSF13 and interneuron markers and the positive correlation with spine markers could suggest a pleiotropic effect of TNFSF13 in the CNS. Consistent with the notion that TNFSF13 may lead to growth inducing events in the dendritic spine rather than solely cell-death related events, FAS receptor activation has been observed to induce stem cell proliferation, differentiation [66] and dendritic branching [67] in the CNS. Our data support the possibility that FAS receptor activation by TNFSF13 in adult cortex leads to activation of alternate intracellular signaling pathways, which have opposing effects on interneurons and pyramidal cells. Another cytokine, interleukin-1β, has been shown to activate excitatory pyramidal neurons and inhibit GABA neurons, leading to an overall increase in excitatory tone and seizure activity [68]. This is consistent with recent work by Behrens and colleagues [18], [69] demonstrating that treatment with the NMDA antagonist, ketamine, results in increased interleukin-6 production, which in turn leads to reduced nicotinamide adenine dinucleotide phosphate (NADPH) oxidase levels and to loss of interneuron phenotype analogous to that observed in patients with schizophrenia. Furthermore, increased activity of NADPH oxidase has previously been shown to result in decreased intracellular pH [70], a proxy for levels of oxidative stress in the measured tissue [71]. It may therefore be that TNFSF13 acts not as an apoptosis inducer, but in the role of a cytokine able to differentially modulate neuron physiology in response to perturbations in the adult CNS. An alternative explanation for the positive correlation beween TNFSF13 and PPP1R9B is that PPP1R9B mRNA expression levels may not directly reflect spine number, but rather spine modifyability. Indeed, young rodents lacking spinophilin (PPP1R9B) have increased spine numbers suggesting more stability but less plasticity [72]. Therefore, the correlation between increased TNFSF13 expression with increased PPP1R9B that we find might reflect less stability of synaptic spines, which in the context of schizophrenia could be detrimental to spine density.

Our study of gene expression changes was limited by restricting it to qRT-PCR measures of mRNA transcripts only. Whether changes observed at the transcript level are reflected in changes in protein levels require further investigation. By focusing on transcripts that had showed expression change by array analysis, we also limited our study in scope. It is possible that other transcripts in the cell death pathway have altered expression levels. However, the main effector molecules in the FAS receptor pathway are caspases, proteases which are regulated at the level of protein cleavage, and therefore better studied at the protein level. Future investigations of the cell death pathway in schizophrenia should include investigation of caspase-3, -6 and -9 levels in addition to the TNFSF13 ligand and FAS receptor.

In conclusion, a broad-spectrum microarray inquiry revealed a selective alteration in the gene expression of molecules related to the TNFSF13 cell death pathway. The microarray-identified alteration in TNFSF13 mRNA was confirmed using qRT-PCR and replicated in an independent brain tissue collection. The alteration in TNFSF13 gene expression showed relative disease specificity, as it was observed in the schizophrenia and not the bipolar disorder cases and showed relative brain region specificity in schizophrenia, as it was observed in the DLPFC and not in the OFC. Of note, we found TNFSF13 mRNA expression in the DLPFC was significantly positively correlated with the spine marker, PPP1R9B (spinophilin) and significantly negatively correlated with mRNA expression of interneuron markers, parvalbumin and somatostatin, the latter being influenced by tissue pH. One explanation for these inverse relationships with inhibitory and excitatory neuronal markers is that the cytokine actions rather than the apoptotic actions of TNFSF13 are mainly contributing to the observed pathology in schizophrenia. We propose a model where TNFSF13, like the interleukins, can increase excitatory pyramidal neuron activity, indexed by increased PPP1R9B, and decrease interneuron health, indexed by tissue pH-dependent decreased expression of interneuron markers, parvalbumin and somatostatin.

## Materials and Methods

### *In silico* pathway analysis of the existing SMRI Array database

To identify candidate cell death pathways, the existing SMRI Array database was searched for the keywords “apoptosis” and “death”. The SMRI Array database consists of results of 6 microarray studies of 35 people with schizophrenia, 35 people with bipolar illness and 35 controls (Array collection) and 6 microarray studies of 15 people with schizophrenia, 15 people with bipolar illness, 15 people with depression and 15 controls (Consortium collection) [48]. While studies mostly focus on prefrontal cortex (BA8/9/10/46), it also includes two studies of the cerebellum. The database reports the fold change and significance for the Consortium collection and the Array collection separately and combined. We relied on the combined data for determining significantly changed gene expressions. The fold change reported in the Array database did not control for potentially confounding factors such as age and tissue pH, though these factors were independently explored for each transcript. For a detailed decription of how the cross-study analysis was performed, see Higgs et al. [48]. A heat map ranking pathways by GO term enrichment was also consulted. These database resources are available to researchers at www.stanleygenomics.org.

### Human postmortem tissue studies

#### Ethics Statement

All research was approved by and conducted under the guidelines of the Human Research Ethics Committee at the University of New South Wales (HREC 07261).

#### Subjects

For the DLPFC, samples from two postmortem brain tissue collections were analysed. The Australian TRC collection [73] consisted of 37 patients with schizophrenia/schizoaffective disorder and 37 unaffected controls. The SMRI Array collection consisted of 35 patients with schizophrenia, 31 patients with bipolar disorder and 34 unaffected controls. RNA extracted from the lateral OFC was obtained from the same subjects within the SMRI Array collection that provided RNA from the DLPFC (one case in the schizophrenia group was excluded because of technical problems). RNA obtained from the SMRI tissues, Maryland, USA (http://www.stanleyresearch.org/dnn/BrainResearchCollection/ArrayCollection/tabid/89/Default.aspx) was transported to Sydney, Australia on dry ice.

Demographic and clinical variables for the SMRI and TRC collections of tissue, separately and combined, are detailed in Tables S2 and S3 and Table 4. Patient and control groups did not differ according to age, sex, brain hemisphere or postmortem delay, except for a significantly greater number of females in the bipolar group in the SMRI collection. Within the SMRI collection the schizophrenia and bipolar groups had significantly lower tissue pH than the unaffected control group. The schizophrenia groups in both the SMRI and NSW TRC collections had a significantly greater proportion of patients who smoked at the time of death, and who died from suicide.

**Table 4:** Demographic and clinical variables of the combined SMRI and NSW TRC collection.

	Control	Schizophrenia	Statistics
N	71	72	
Race (A/B/C/D)^a^	70/1/0	70/1/1	
Age (years)	47.6±12.2	47.1±12.5	t(141) = 0.274, p = 0.785
Sex (male/female)	55/16	50/22	X^2^(1) = 1.179, p = 0.278
Hemisphere (left/right)	30/41	37/35	X^2^(1) = 1.198, p = 0.274
Postmortem delay	27.04±12.15	29.89±14.63	t(141) = −1.267, p = 0.207
Tissue pH	6.64±0.28	6.55±0.28	t(141) = 1.932, p = 0.055
Death by suicide (yes/no)	0/71	15/57	X^2^(1) = 16.525, p = 0.000
Smoking status (yes/no)	18/21	46/11	X^2^(1) = 12.437, p = 0.000
Duration of illness (years)	-	24.54±12.50	
Age of onset (years)	-	22.53±6.15	
Medication (lifetime chlorpromazine)	-	6,129,700±6,893,220	

The combined SMRI and NSW TRC collections were utilized for analysis of gene expression in the DLPFC only. Data are provided as means ± the standard deviation or number of individuals in each category.

a A/B/C/D: European/Asian/Hispanic.

#### RNA extraction and qRT-PCR

For both collections, RNA extraction was carried out using TRIzol Reagent (Invitrogen Life Sciences, Melbourne, VIC, Australia). cDNA was generated using a SuperScript First-Strand Synthesis kit (Invitrogen, Carlsbad, CA, USA) as described in Weickert *et al.* [73].

Expression levels of 12 transcripts (see Table 1) were used in this study, including 5 apoptosis-related transcripts: TNFSF13; FAS receptor; BID; two transcript variants (pan and long) of CASP8 and FADD-like apoptosis regulator (CFLAR); 2 spine marker mRNAs: discs, large homolog 4 (DLG4, also known as PSD-95); and protein phosphatase 1, regulatory (inhibitor) subunit 9B (PPP1R9B, also known as spinophilin); 2 previously measured mRNAs for inhibitory markers parvalbumin and somatostatin [41]; and 3 housekeeper genes: β-actin; TATA box binding protein; and ubiquitin C. These were measured by qRT-PCR using published methods [73]. Samples were measured in triplicate and measurement outliers removed. The relative quantities of mRNA levels were calculated using the deltadelta Ct method, normalizing to the geomean Ct levels of housekeeping genes. The amplification efficiency for the three housekeeping genes ranged from 64 to 89%, whereas the amplification efficiency for test genes ranged from 60 to 104%.

### Cell culture studies

We found an unexpected strong negative correlation between gene expression of TNFSF13 and tissue pH and explored whether a low pH caused increased TNFSF13 or if increased TNFSF13 could cause decreased pH. Since TNFSF13 and FAS receptor can be synthesized in glia, the U-87 MG human glioblastoma (ACTT Catalogue Number HTB-14) cell line was cultured under standard conditions (37°C, 5% CO_2_) in Dulbecco's modified Eagle media with high glucose supplemented with 10% fetal bovine serum. Cells were seeded in culture plates at 1.25×10^6^ cells/mL 24 hours prior to commencement of assays.

#### Effect of reduced intra-cellular pH on TNFSF13 expression

U-87 MG cells were plated into 12-well plates. On the day of the assay, 50% of the culture media was replaced with buffers containing 120 mM potassium phosphate, 20 mM sodium chloride at pH 7.3, 6.9 and 6.4. These buffers also contained 1 µM nigericin (0.5 µM final concentration; Invitrogen Life Sciences, Australia) which rapidly decreases the intracellular pH to that of the culture media. Thirty minutes, 3, 12 and 24 hours later, triplicate cultures at each pH were washed twice in PBS and RNA extracted using TRIzol reagent according to the manufacturer's instructions. Transcript levels of TNFSF13 and FAS receptor and 3 housekeeper genes – β-actin, TATA box binding protein and ubiquitin C – were determined by qRT-PCR as described above.

#### Effect of TNFSF13 on intra-cellular pH

U-87 MG cells were plated into 96-well plates. Human recombinant TNFSF13 (Catalogue Number 884-AP, RD Systems) was added (in 50% fresh media) at final concentrations of 0, 1 and 100 ng TNFSF13/mL media 48, 24 and 12 hours prior to pH determination. At time zero (48 hours after assay commencement), all wells were quickly washed in 37°C PBS, pH 7.4 followed by incubation for 30 minutes at 37°C with 10 µM 2′,7′-bis-(2-carboxyethyl)-5-(and-6)-carboxyfluorescein, acetoxymethyl ester (BCECF-AM: Invitrogen Life Sciences, Australia) in PBS. Wells were then washed once with fresh, cold PBS and the 96-well culture plate placed on ice. Following 488 nm excitation, the 525/610 fluorescence ratio, which is indicative of intracellular pH [74], was determined using a plate reader (POLARstar, BMG Labtech). Results are presented as percent change in 525/610 ratio from that of control cells.

### Statistical analysis

Statistical analyses were conducted using SPSS version 17.0. Population outliers were determined using the Grubb's test. Data were analyzed using parametric and non-parametric tests as appropriate to determine group differences in gene expression levels, and to examine if gender, hemisphere, smoking status, antidepressant medication or peri-mortem factors were related to gene expression levels. We correlated gene expression levels for each diagnostic group with continuous demographic (age, pH, PMI) and clinical (age of onset, duration of illness, daily chlorpromazine equivalent dose, last reported chlorpromazine equivalent dose, lifetime equivalent chlorpromazine dose) variables. Statistical significance was set at p≤0.05 with tests being one-tailed (when measuring gene expression changes predicted by the array results) or two-tailed (when assessing unpredicted relationships). Effect sizes were calculated for diagnostic effects according to Field [75]. High expression of TNFSF13 and FAS receptor was defined as deltadelta Ct values greater than the median plus 1.25 times the interquartile range for the unaffected control group. We determined the values for the normal distribution of TNFSF13 and FAS receptor mRNA expression by reiterative step-wise removal of the highest expressor and testing of normality using the Kolmogorov-Smirnow statistics until such point where the significance value was greater than 0.05. Linear regression was carried out with somatostatin, parvalbumin, DLG4 or PPP1R9B transcript levels as the dependent variable, specifying TNFSF13 alone or in combination with tissue pH as independent variables.

## Supporting Information

Figure S1 **Gene expression levels of TNFSF13-FAS receptor pathway genes of interest relative to three housekeeping genes in dorsolateral prefrontal cortex from patients with schizophrenia.** cDNA samples obtained from the SMRI (Panel A, C, E, G, I) and NSW TRC (Panel B, D, F, H, J) collections from individuals with schizophrenia and unaffected controls were subjected to qRT-PCR. Expressions of TNFSF13 (A, B), FAS receptor (C, D), CFLAR_pan_ (E, F), CFLAR_long_ (G, H), and BID (I, J) relative to three housekeeping genes (β-actin, TATA box binding protein and ubiquitin C) were calculated using the deltadelta Ct method. Horizontal lines indicate the population median, except for panel (E) where they indicate the mean as those data were normally distributed. * p<0.05.
(TIF)

Table S1 **Pathway analysis of the SMRI Array database identified 17 apoptotic pathways of potential interest.**
(DOC)

Table S2 **Demographic and clinical variables of groups included in the SMRI collection.**
(DOC)

Table S3 **Demographic and clinical variables of groups included in the NSW TRC collection.**
(DOC)
