# Supplementary figures and images for: Gene Expression Analysis Implicates a Death Receptor Pathway in Schizophrenia Pathology
Source: PLoS One. 2012 Apr 24;7(4):e35511. doi: 10.1371/journal.pone.0035511 (PMC3335850; doi:10.1371/journal.pone.0035511)

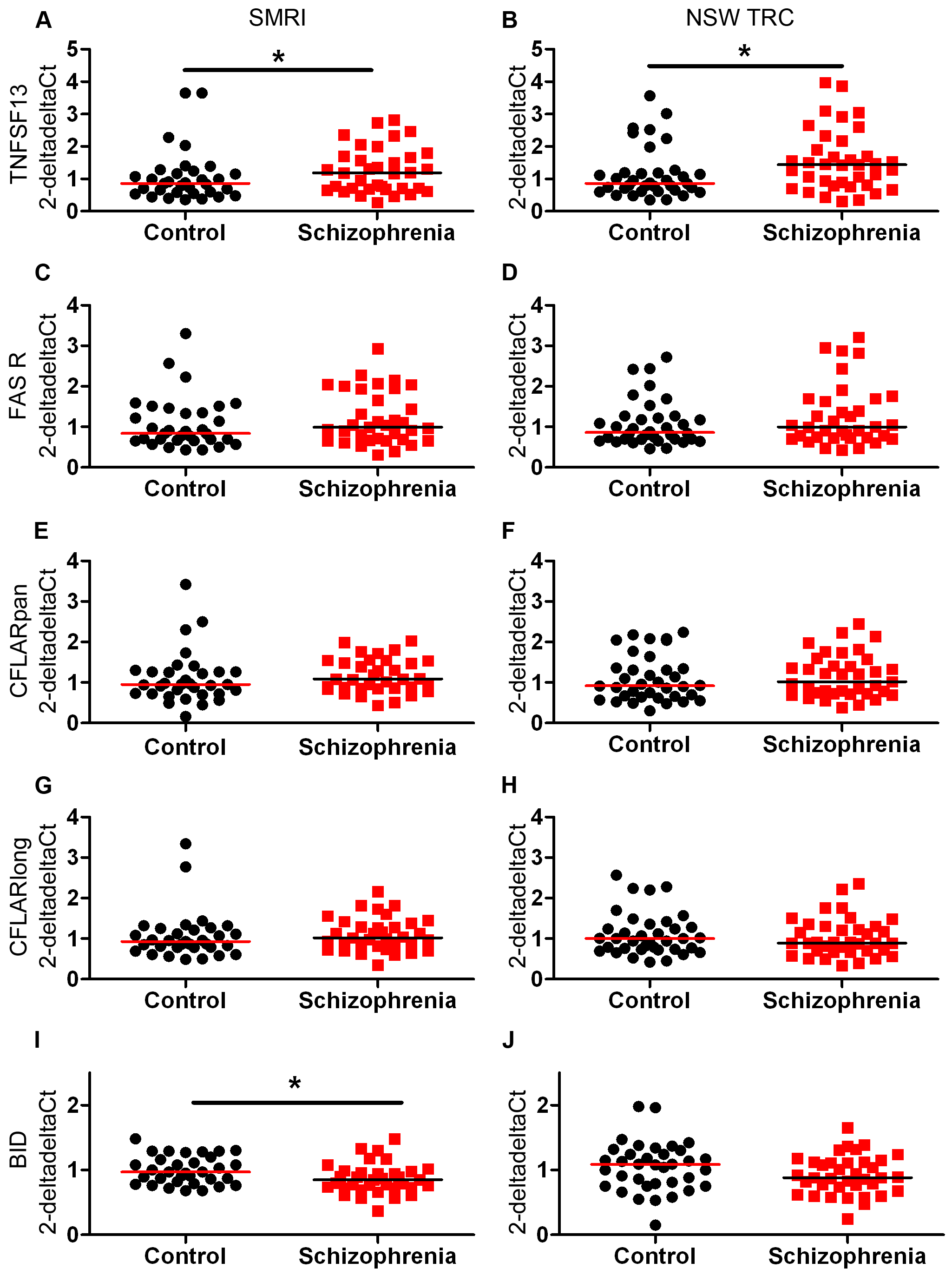

Supplement: Figure S1 — Gene expression levels of TNFSF13-FAS receptor pathway genes of interest relative to three housekeeping genes in dorsolateral prefrontal cortex from patients with schizophrenia. cDNA samples obtained from the SMRI (Panel A, C, E, G, I) and NSW TRC (Panel B, D, F, H, J) collections from individuals with schizophrenia and unaffected controls were subjected to qRT-PCR. Expressions of TNFSF13 (A, B), FAS receptor (C, D), CFLARpan (E, F), CFLARlong (G, H), and BID (I, J) relative to three housekeeping genes (β-actin, TATA box binding protein and ubiquitin C) were calculated using the deltadelta Ct method. Horizontal lines indicate the population median, except for panel (E) where they indicate the mean as those data were normally distributed. * p<0.05. (TIF) [file pone.0035511.s001.tif]
