# Supplementary material for: Gene Expression Analysis Implicates a Death Receptor Pathway in Schizophrenia Pathology
Source: PLoS One. 2012 Apr 24;7(4):e35511. doi: 10.1371/journal.pone.0035511 (PMC3335850; doi:10.1371/journal.pone.0035511)
Supplement: Table S1 — Pathway analysis of the SMRI Array database identified 17 apoptotic pathways of potential interest. (DOC) [file pone.0035511.s002.doc]

| **Pathway** | **Genes changed/**  **genes in pathway** | **Changed mRNA transcripts** |
| --- | --- | --- |
| Induction of apoptosis through DR3 and DR4/5 death receptors | 5/29 | ↓ BH3 interacting domain death agonist (BID), p=0.0288  ↑ Casp8 and FADD-like apoptosis regulator (CFLAR), p=0.0343  ↑ Lamin A/C, p=0.0001  ↑ TNF(ligand) superfamily, member 13 (TNFSF13), p=0.0114  ↑ TNF receptor superfamily member 6 (FAS), p=0.0014 |
| FAS signaling pathway | 4/30 | ↑ Casp8 and FADD-like apoptosis regulator (CFLAR), p=0.0343  ↑ Lamin A/C, p=0.0001  ↑ TNF receptor superfamily member 6 (FAS), p=0.0014  ↓ Mitogen-activated protein kinase kinase 4, p=0.0181 |

| Apoptosis | 5/84 | ↓ BH3 interacting domain death agonist (BID), p=0.0288  ↑ Casp8 and FADD-like apoptosis regulator (CFLAR), p=0.0343  ↓ Interleukin-1 receptor associated kinase 1, p=0.0257  ↓ Protein kinase cAMP-dependent regulatory type 1 alpha, p=0.0468  ↑ TNF receptor superfamily member 6 (FAS), p=0.0014 |
| --- | --- | --- |
| TSP-1 induced apoptosis in microvascular endothelial cell | 1/7 | ↑ FYN oncogene related to SRC, FGR, YES, p=0.04 |
| HIV induced T cell apoptosis | 1/10 | ↑ TNF receptor superfamily member 6 (FAS), p=0.0014 |
| Neuropeptides VIP and PACAP inhibit the apoptosis of activated T cells | 1/15 | ↑ v-myc myelocytomatosis viral oncogene homolog (avian), p=0.0391 |
| Role of mitochondria in apoptotic signaling | 1/21 | ↓ BH3 interacting domain death agonist (BID), p=0.0288 |
| Apoptotic signaling in response to DNA damage | 1/21 | ↓ BH3 interacting domain death agonist (BID), p=0.0288 |
| Caspase cascade in apoptosis | 1/23 | ↑ Lamin A/C, p=0.0001 |
| Apoptotic DNA fragmentation and tissue homeostasis | 0/9 |  |
| Free radical induced apoptosis | 0/9 |  |
| Granzyme A mediated apoptosis pathway | 0/11 |  |
| Role of MEF2D in T-cell apoptosis | 0/11 |  |
| Role of nicotinic acetylcholine in the regulation of apoptosis | 0/14 |  |
| Multiple antiapoptotic pathways from IGF-1R signaling lead to BAD phosphorylation | 0/18 |  |
| PTEN dependent cell cycle arrest and apoptosis | 0/18 |  |
