# Supplementary material for: Gene Expression Analysis Implicates a Death Receptor Pathway in Schizophrenia Pathology
Source: PLoS One. 2012 Apr 24;7(4):e35511. doi: 10.1371/journal.pone.0035511 (PMC3335850; doi:10.1371/journal.pone.0035511)
Supplement: Table S2 — Demographic and clinical variables of groups included in the SMRI collection. (DOC) [file pone.0035511.s003.doc]

|  | Control | Schizophrenia | Bipolar disorder | Statistics |
| --- | --- | --- | --- | --- |
| N | 34 | 35 | 31 |  |
| Race (A/B/C/D)^a^ | 34/0/0/0 | 34/0/1/0 | 31/0/0/1 |  |
| Age (years) | 43.8 ± 7.4 | 42.6 ± 8.5 | 44.94 ± 11.0 | F(2,97)=0.571, p=0.567 |
| Sex (male/female) | 25/9 | 26/9 | 15/16 | Χ^2^(2)=6.215, p=0.045; CON vs SZ Χ^2^(1)=0.005, p=0.943; CON vs BPD Χ^2^(1)=4.331, p=0.037 |
| Hemisphere (left/right) | 16/18 | 17/18 | 17/14 | Χ^2^(2)=0.437, p=0.804 |
| Postmortem delay | 29.47 ± 13.05 | 31.40 ± 15.54 | 36.61 ± 18.12 | F(2,97)=1.796, p=0.171 |
| Tissue pH | 6.61 ± 0.27 | 6.47 ± 0.24 | 6.46 ± 0.28 | F(2,97)=3.456, p=0.035; CON vs SZ t(67)=2.269, p=0.027; CON vs BPD t(63)=2.252, p=0.028 |
| Death by suicide (yes/no) | 0/34 | 7/28 | 14/17 | Χ^2^(2)=19.967, p=0.000; CON vs SZ Χ^2^(1)=7.568, p=0.006; CON vs BPD Χ^2^(1)=19.570, p=0.000 |
| Smoking status (yes/no) | 9/9 | 23/4 | 14/6 | Χ^2^(2)=6.472, p=0.039; CON vs SZ Χ^2^(1)=6.508, p=0.011; CON vs BPD Χ^2^(1)=1.586, p=0.208 |
| Alcohol use (1/2/3)^b^ | 29/3/3 | 17/6/12 | 11/10/10 | Χ^2^(4)=19.588, p=0.001; CON vs SZ Χ^2^(2)=11.261, p=0.004; CON vs BPD Χ^2^(2)= 17.101, p=0.000 |
| Substance abuse (1/2/3) ^b^ | 33/0/1 | 18/6/9 | 14/10/8 | Χ^2^(4)=24.814, p=0.000; CON vs SZ Χ^2^(2)=16.801, p=0.000; CON vs BPD Χ^2^(2)=23.086, p=0.000 |
| Duration of illness (years) | - | 21.29 ± 10.15 | 20.16 ± 9.89 |  |
| Age of onset (years) | - | 21.29 ± 6.07 | 24.77 ± 8.95 |  |
| Medication (lifetime chlorpromazine) | - | 4,250,200 ± 5,016,770 | 503,438 ± 1,175,620 |  |
| Mood stabiliser (yes/no) | - | 11/24 | 22/10 |  |

The SMRI collection was utilized for analysis of gene expression in the dorsolateral prefrontal cortex and the orbital frontal cortex. Data are provided as means ± the standard deviation or number of individuals in each category.

^a^ A/B/C/D: European/Asian/Hispanic/Native American.

^b^ 1/2/3: no or social only use/ moderate-heavy past use/ moderate-heavy present use
