# Supplementary material for: Gene Expression Analysis Implicates a Death Receptor Pathway in Schizophrenia Pathology
Source: PLoS One. 2012 Apr 24;7(4):e35511. doi: 10.1371/journal.pone.0035511 (PMC3335850; doi:10.1371/journal.pone.0035511)
Supplement: Table S3 — Demographic and clinical variables of groups included in the NSW TRC collection. (DOC) [file pone.0035511.s004.doc]

|  | Control | Schizophrenia | Statistics |
| --- | --- | --- | --- |
| N | 37 | 37 |  |
| Race (A/B/C/D)^a^ | 36/1/0/0 | 36/1/0/0 |  |
| Age (years) | 51.1 ± 14.6 | 51.3 ± 14.1 | t(72)=-0.057, p=0.955 |
| Sex (male/female) | 30/7 | 24/13 | Χ^2^(1)=2.467, p=0.116 |
| Hemisphere (left/right) | 14/23 | 20/17 | Χ^2^(1)=1.959, p=0.162 |
| Postmortem delay | 24.80 ± 10.97 | 28.46 ± 13.77 | t(72)=-1.264, p=0.210 |
| Tissue pH | 6.66 ± 0.30 | 6.61 ± 0.30 | t(72)=0.641, p=0.523 |
| Agonal status (1/2/3)^b^ | 14/20/3 | 3/26/8 | Χ^2^(2)=10.173, p=0.006 |
| Death by suicide (yes/no) | 0/37 | 8/29 | Χ^2^(1)=8.970, p=0.003 |
| Smoking status (yes/no) | 9/12 | 23/7 | Χ^2^(1)=6.041, p=0.014 |
| Duration of illness (years) | - | 27.62 ± 13.82 |  |
| Age of onset (years) | - | 23.70 ± 6.07 |  |
| Medication (lifetime chlorpromazine) | - | 7,910,000 ± 7,953,630 |  |
| Antipsychotic agent prescribed^c^ |  | Risperidone (13), fluphenazine decanoate (7), olanzapine (6), clozapine (6), flupenthixol (3), haloperidol (2), amisulpride (2), zuclopentixol (2), chlorpromazine (1), quetiapine (1), stelazine (1) |  |

The NSW TRC collection was utilized for analysis of gene expression in the dorsolateral prefrontal cortex only. Data are provided as means ± the standard deviation or number of individuals in each category.

^a^ A/B/C/D: European/Asian/Hispanic/Native American.

^b^ Agonal status was assessed based on a 3 point scale: excellent (1), good (2) and poor (3). Donors with a short agonal status and good physical condition were rates “1’, those with a moderate agonal phase and average physical condition were rated “2”, and donors with a longer agonal phase, and/or poor physical condition prior to death were rated “3’ as detailed in Weickert et al. (2010, Aust N Z J Psychiatry 44:59-70).

^c^The total number of prescriptions are greater than 37 as some individuals were prescribed more than one antipsychotic medication.
